# Pulmonary abnormality screening on chest x-rays from different machine specifications: a generalized AI-based image manipulation pipeline

**DOI:** 10.1186/s41747-023-00386-1

**Published:** 2023-11-09

**Authors:** Heejun Shin, Taehee Kim, Juhyung Park, Hruthvik Raj, Muhammad Shahid Jabbar, Zeleke Desalegn Abebaw, Jongho Lee, Cong Cung Van, Hyungjin Kim, Dongmyung Shin

**Affiliations:** 1 Artificial Intelligence Engineering Division, RadiSen Co., Ltd, Seoul, Korea; 2 Laboratory for Imaging Science and Technology, Department of Electrical and Computer Engineering, Seoul National University, Seoul, Korea; 3 Department of Radiology, National Lung Hospital, Hanoi, Vietnam; 4 Department of Radiology, Seoul National University Hospital, Seoul, Korea

**Keywords:** Artificial intelligence, Chest radiography, Computer-aided classification, Deep learning, Image postprocessing (computer-assisted)

## Abstract

**Background:**

Chest x-ray is commonly used for pulmonary abnormality screening. However, since the image characteristics of x-rays highly depend on the machine specifications, an artificial intelligence (AI) model developed for specific equipment usually fails when clinically applied to various machines. To overcome this problem, we propose an image manipulation pipeline.

**Methods:**

A total of 15,010 chest x-rays from systems with different generators/detectors were retrospectively collected from five institutions from May 2020 to February 2021. We developed an AI model to classify pulmonary abnormalities using x-rays from a single system. Then, we externally tested its performance on chest x-rays from various machine specifications. We compared the area under the receiver operating characteristics curve (AUC) of AI models developed using conventional image processing pipelines (histogram equalization [HE], contrast-limited histogram equalization [CLAHE], and unsharp masking [UM] with common data augmentations) with that of the proposed manipulation pipeline (XM-pipeline).

**Results:**

The XM-pipeline model showed the highest performance for all the datasets of different machine specifications, such as chest x-rays acquired from a computed radiography system (*n* = 356, AUC 0.944 for XM-pipeline *versus* 0.917 for HE, 0.705 for CLAHE, 0.544 for UM, *p* $\le$ 0.001, for all) and from a mobile x-ray generator (*n* = 204, AUC 0.949 for XM-pipeline *versus* 0.933 for HE, *p* = 0.042, 0.932 for CLAHE (*p* = 0.009), 0.925 for UM (*p* = 0.001).

**Conclusions:**

Applying the XM-pipeline to AI training increased the diagnostic performance of the AI model on the chest x-rays of different machine configurations.

**Relevance statement:**

The proposed training pipeline would successfully promote a wide application of the AI model for abnormality screening when chest x-rays are acquired using various x-ray machines.

**Key points:**

• AI models developed using x-rays of a specific machine suffer from generalization.

• We proposed a new image processing pipeline to address the generalization problem.

• AI models were tested using multicenter external x-ray datasets of various machines.

• AI with our pipeline achieved the highest diagnostic performance than conventional methods.

**Graphical Abstract:**

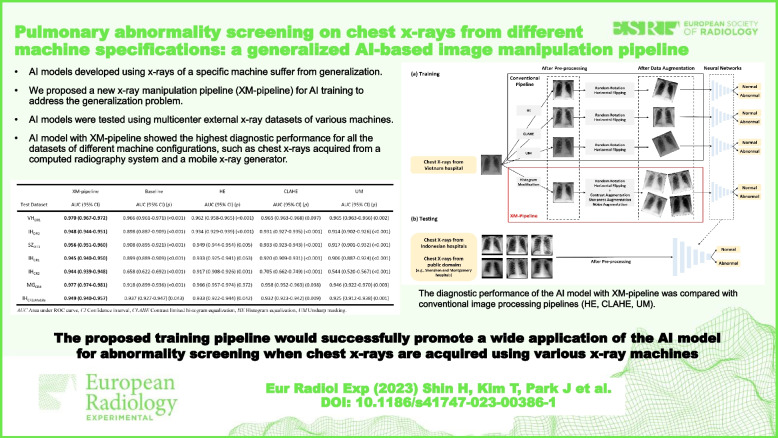

**Supplementary Information:**

The online version contains supplementary material available at 10.1186/s41747-023-00386-1.

## Background

Chest x-ray is the most common medical imaging exam to screen patients suspected of having pulmonary abnormalities and diseases. Due to its utility, many deep learning-based artificial intelligence (AI) methods have been proposed, such as detecting pneumonia, tuberculosis, and COVID-19 [1–5]. Also, some studies have explored the potential applications of those methods in clinical environments, including shortening turnaround time, increasing reading efficiency, and reducing misinterpretation [6–10].

Chest x-ray images have their unique characteristics depending on the specifications of x-ray machines which are broadly composed of detectors (*e.g.*, computed radiography (CR) and digital radiography (DR)) and generators (*e.g.*, mobile or stationary). For example, chest x-ray images from DR detectors typically show better image quality than those from CR detectors at the same dose level [11]. In addition, chest x-ray images from mobile x-ray machines usually have higher noise than those from stationary machines due to the limited maximum power of generators [12, 13].

However, many AI methods for chest x-ray abnormality or disease classification have not strictly investigated their method’s stability for chest x-rays of different x-ray machine specifications, even though supervised-trained AI algorithms presumably have a high bias toward the training dataset [14]. This bias results in the degraded diagnostic performance of the methods when applied to chest x-rays with different characteristics from training data (*i.e.*, x-rays of unseen machines during training), limiting the clinical utility in the real world [15–17].

In this study, we propose an x-ray manipulation pipeline (XM-pipeline) that combines a set of image pre-processing and data augmentation techniques to overcome the AI model’s bias toward the training dataset from a single x-ray machine. We carefully designed the XM-pipeline to incorporate the hardware-related changes in x-ray images during AI training. To validate the effectiveness of the XM-pipeline, we trained AI models using the XM- and conventional pipelines. Then, we compared their diagnostic performance based on multiple test datasets of different machine specifications.

## Methods

### Chest x-ray image collection and annotation

**Fig. 1:**
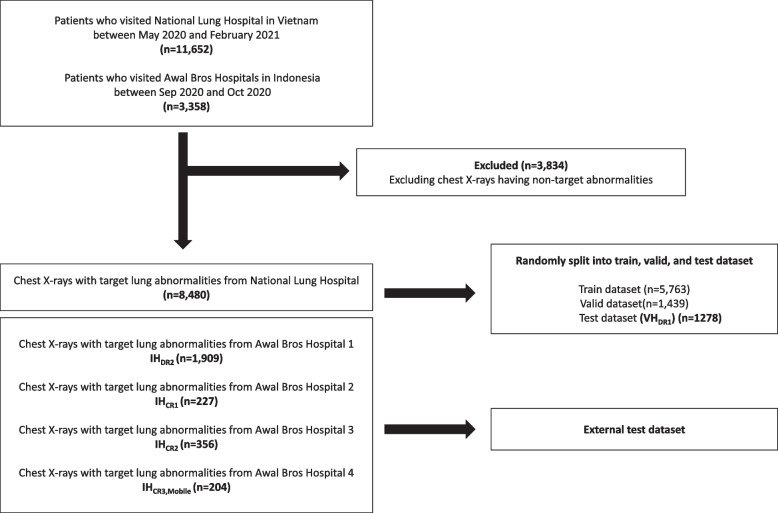
Data flow diagram of our retrospective study

In our retrospective study, we collected chest x-ray images (digital imaging and communications in medicine [DICOM] format) from Vietnam and Indonesian hospitals (Fig. 1). A total of 11,652 chest x-ray images of symptomatic patients who visited the National Lung Hospital in Vietnam for tertiary care were acquired between May 2020 and February 2021. Also, from Awal Bros hospitals located in four different areas of Indonesia, 3,358 chest x-ray images of asymptomatic individuals who underwent medical checkups were collected between September 2020 and October 2020. Each hospital used different x-ray machine specifications (*i.e.*, CR or DR detectors of different vendors with stationary or mobile generators; see Table 1 and Supplementary Table S1 for details). The institutional review board of each participating institution approved this study.

**Table 1:** Summary of the test datasets used in our retrospective study

Detector	Generator	Test dataset	Institution	Country	Radiologist for annotation	Number of patients	Number of abnormal x-rays	Number of normal x-rays	Sex (male/female/unknown)	Age (mean $\pm$ standard deviation)
DR	Stationary	VH_DR1_	National Lung Hospital	Vietnam	MD_1_	1,278	1,013	265	1,027/251/0	34.66 $\pm$ 28.80
		IH_DR2_	Awal Bros Hospital 1	Indonesia	MD_1_	1,909	330	1,579	813/610/486	37.21 $\pm$ 24.09
		SZ_DR3_	Shenzhen Hospital	China	MD_2_	662	336	326	460/202/0	35.57 $\pm$ 14.71
CR		IH_CR1_	Awal Bros Hospital 2	Indonesia	MD_1_	227	58	169	84/71/72	42.68 $\pm$ 14.74
		IH_CR2_	Awal Bros Hospital 3	Indonesia	MD_1_	356	82	274	156/200/0	40.86 $\pm$ 16.45
		MG_CR4_	Montgomery Hospital	USA	MD_3_	138	58	80	63/74/1	40.11 $\pm$ 18.79
	Mobile	IH_CR3,Mobile_	Awal Bros Hospital 4	Indonesia	MD_1_	204	85	119	129/74/1	44.39 $\pm$ 13.14

A radiologist with 30 years of experience (MD_1_) reviewed all the chest x-rays from Vietnam and Indonesian hospitals. The presence of common pulmonary abnormalities (*i.e.*, target abnormalities: atelectasis, consolidation/ground glass opacity, fibrotic sequelae, nodule/mass, and pneumothorax) for each chest x-ray image was confirmed by the radiologist using a web-based annotation tool (Label Studio version 1.6) [18]. Based on the radiologist’s annotations, we excluded 3,834 chest x-ray images with non-target abnormalities (Fig. 1; see Supplementary Table S2 for distribution of target abnormalities). Then, the chest x-ray images from the Vietnam hospital were randomly split into training (5,763 chest x-rays), validation (1,439 chest x-rays), and internal testing datasets (1,278 chest x-rays; VH_DR1_ in Table 1). Also, the chest x-ray images from Indonesian hospitals were separated into four datasets according to each x-ray machine and hospital as external testing datasets (IH_DR2_, IH_CR1_, IH_CR2_, and IH_CR3,Mobile_ in Table 1). To check the potential reproducibility issue of the annotations, we also invited three more radiologists (12 years of experience on average; MD_2_, MD_3_, and MD_4_) to annotate some chest x-rays of VH_DR1_ (100 normal and 100 abnormal x-rays) and calculated Cohen’s kappa scores between MD_1_ and the others. This results in high Cohen’s kappa scores (0.84 for MD_2_, 0.87 for MD_3_, and 0.81 for MD_3_), which means the annotations of MD_1_ are highly consistent and reproducible by the other radiologists.

In addition to the collected datasets, we utilized five publicly available chest x-ray datasets for AI evaluation: Two datasets for tuberculosis detection were Shenzhen [19] and Montgomery datasets (SZ_DR3_ and MG_CR4_ in Table 1; Portable Network Graphics format) [19]. Three large public datasets included CheXpert [20], ChestX-Det10 [21], and RSNA-Pneumonia [22] (Portable Network Graphics or Joint Photographic Experts Group format; see Supplementary Note 1).

**Fig. 2:**
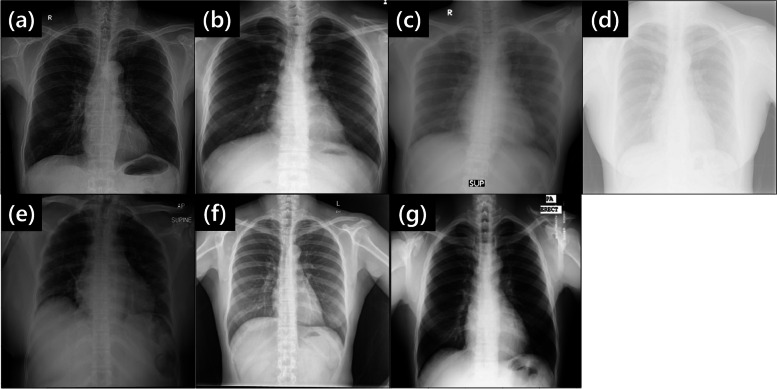
Example chest x-ray images of different x-ray machine specifications: (**a**) VH_DR1_, (**b**) IH_DR2_, (**c**) IH_CR1_, (**d**) IH_CR2_, (**e**) IH_CR3,Mobile_, (**f**) SZ_DR3_, and (**g**) MG_CR4_. Each image reveals unique characteristics (*e.g.*, contrast and noise levels). The images were plotted without adjusting the window level and width except for SZ_DR3_ and MG_CR4_. In other words, raw image data were extracted from DICOM files

Figure 2 shows some example chest x-ray images from the test datasets without adjusting the window level and width (*i.e.*, raw DICOM images), highlighting diverse image characteristics.

### Training AI models

**Fig. 3:**
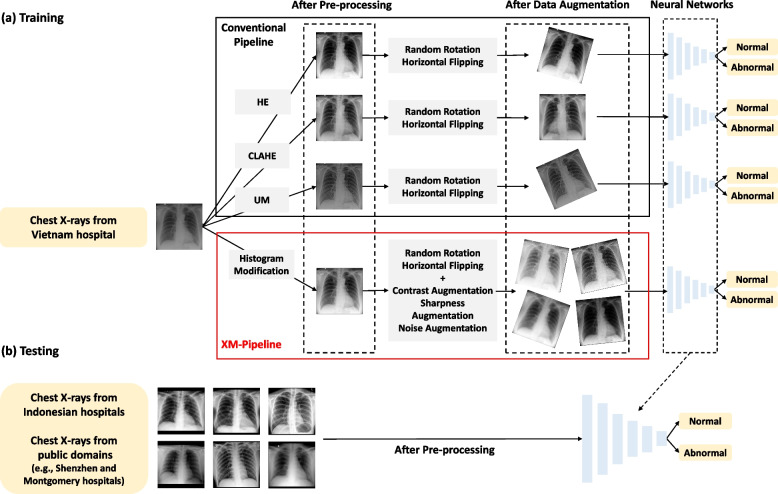
Overview of training and testing phases of the AI models used in this study. **a** Training phase: after cropping the lung regions of chest x-ray images, those images were processed by each pipeline for AI training. The conventional pipelines utilized HE, CLAHE, and UM as pre-processing methods, and random rotation and horizontal flipping as data augmentations. The XM-pipeline includes the histogram modification and three more data augmentation techniques (*i.e.*, contrast, sharpness, and noise augmentations). All networks were trained to classify chest x-rays as normal or abnormal. **b** Testing phase: external test datasets of different x-ray machine specifications were used for AI evaluation. All input x-rays were pre-processed before being fed to the networks. *CLAHE* Contrast-limited histogram equalization, *HE* Histogram equalization, *UM* Unsharp masking

We utilized an EfficientNet-B6 [23] as a neural network architecture and trained five AI models by applying conventional image processing pipelines, the XM-pipeline, and no pipeline (*i.e.*, baseline) (Fig. 3) to classify chest x-ray images as normal (*i.e.*, no target abnormalities) or abnormal (*i.e.*, with at least one target abnormality). Each pipeline is composed of two sub-functions, pre-processing, and data augmentation. We cropped the lung regions of all chest x-ray images before applying the pipelines using an additional network developed by in-house data.

We used Pytorch (Version 1.12.1) and an NVIDIA GeForce RTX 3090 for AI training. We utilized an Adam (learning rate 0.003; batch size 4) [24] as an optimizer. In the AI training phase, we applied a resampling method, which under-sampled the majority class data, to mitigate the data imbalance problem [25]. All chest x-ray images were resized to 512 × 512 after pre-processing.

### Conventional image-processing pipelines

For conventional image pre-processing, we adopted histogram equalization (HE) [26], contrast-limited adaptive histogram equalization (CLAHE) [27], and unsharp masking (UM) [28], which are primarily utilized in chest x-ray AI development [29–32]. Also, as conventional data augmentation techniques, we applied random rotation (degree within [-15, 15]) and horizontal flipping (probability 0.5), which are commonly used in many studies of chest x-ray AI [33–37].

### X-ray manipulation pipeline (XM-pipeline)

**Fig. 4:**
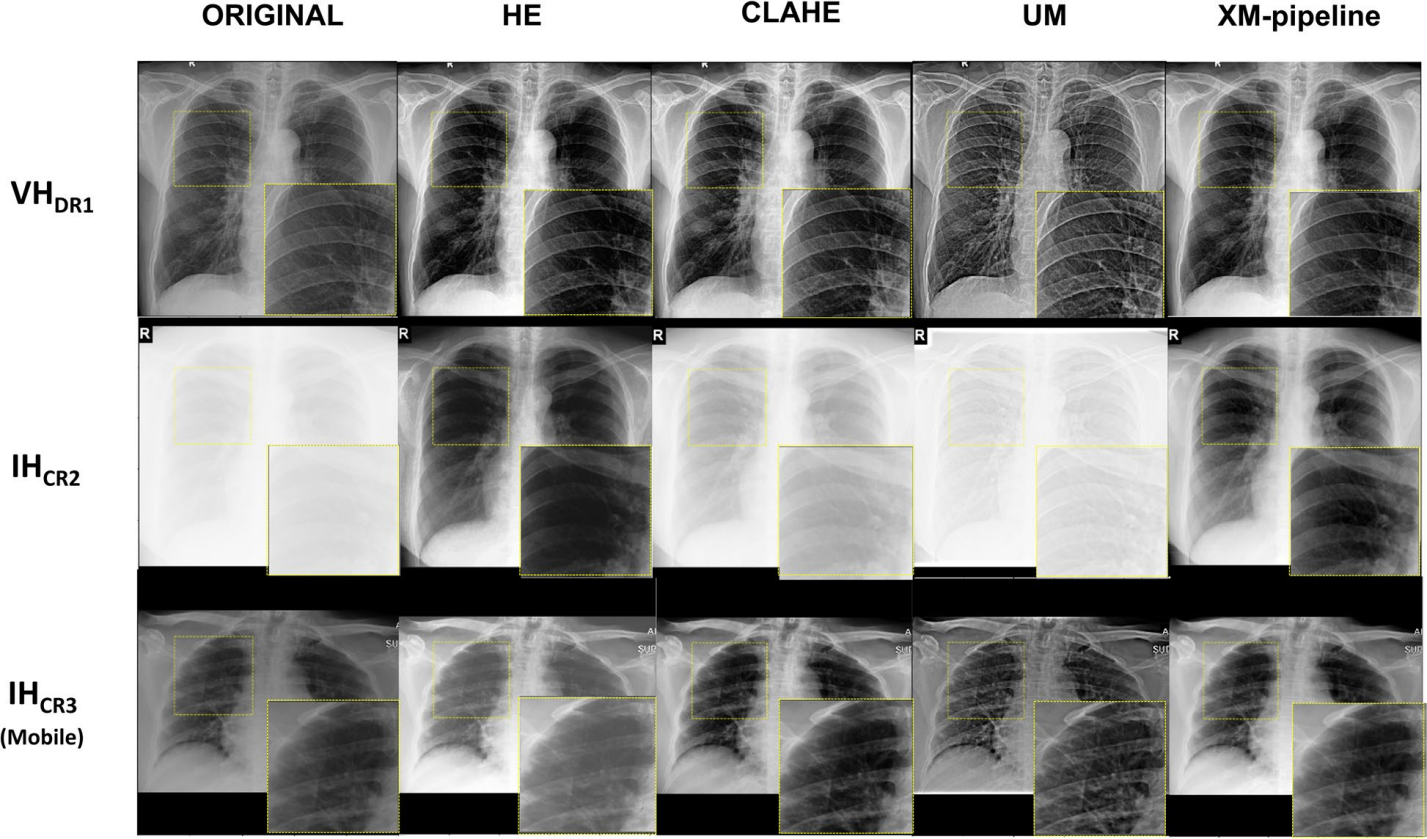
Example chest x-ray images from VH_DR1_, IH_CR2_, and IH_CR3,Mobile_ after applying the conventional pre-processing methods (HE, CLAHE, UM) and the histogram modification in the XM-pipeline: The right upper zones (yellow-dotted boxes) of each image were zoomed in for investigation. *CLAHE* Contrast-limited histogram equalization, *HE* Histogram equalization, *UM* Unsharp masking

In the proposed XM-pipeline, as a pre-processing step, we modified the histogram of each chest x-ray image to normalize its brightness and maximize the information inside the lung region (Supplementary Note 2 for detail). First, we stretched the histogram through an iterative optimization process [38], and to improve the contrast inside the lung region, we changed the minimum intensity of each x-ray image to the minimum intensity inside the lung region [32]. Example chest x-ray images after pre-processing are shown in Fig. 4 (more examples in Supplementary Fig. S1).

The histogram modification was then followed by contrast, sharpness, and noise augmentation techniques (Supplementary Note 3 for detail) in the training phase to mimic the hardware-related changes of chest x-rays. We simulated the contrast change of chest x-ray images depending on the voltage level of an x-ray generator using a gamma correction method [39]. Also, we mimicked the change in the sharpness of x-rays, possibly due to a scattering effect, by applying a Gaussian filter [40]. Finally, to consider the thermal and electronic noises, we added synthetic noise to each chest x-ray image [41].

### Evaluation of AI models

For all the test datasets, the diagnostic performance of each AI model was evaluated by calculating the average area under the curve (AUC) at receiver operating characteristics analysis with 95% confidence intervals (CIs).

The DeLong test [42] was performed to check the statistical significance of the difference in the diagnostic performance of each pair of the AI model with the XM-pipeline and another. We repeated AI training ten times by iterating the random division of training and validation data (5,763 chest x-rays (80%) for training; 1,439 chest x-rays (20%) for validation; 1,278 chest x-rays for internal testing were totally separated; Fig. 1). Each random split of data for each iteration was consistent across the different model settings in Fig. 3. We utilized Fisher’s method to combine *p* values from each iteration and check the statistical significance (*p* < 0.05) for each pair (*i.e.*, XM *versus* another), instead of performing multiple comparisons (*i.e.*, comparisons between all combinations such as HE *versus* CLAHE).

We also checked the stability of each AI model for the changes in the characteristics of x-ray images (*e.g.*, noise injection). We utilized the internal testing dataset (VH_DR1_ in Table 1) to generate x-ray images with different contrast ($\gamma$ = 0.2 to 5.0), sharpness ($s$ = -12 to 12), and noise ($\sigma$ = 0 to 0.1) levels (details in Supplementary Note 3). Then, we fed those images to each AI model and calculated the averaged AUC values depending on the changes in contrast, sharpness, and noise levels. For the AUC calculation, we also repeated AI training ten times.

All statistical analysis was performed using Python packages (scikit-learn (1.2.0) and SciPy (1.7.3)).

## Results

### Diagnostic performance of AI models

The diagnostic performance of the AI models (AUCs and *p* values) is summarized in Table 2. Other evaluation metrics, such as sensitivity and specificity for each AI model, are summarized in Supplementary Table S3. For VH_DR1_, which is the internal test dataset from the same source of the training data, the diagnostic performance of the AI model with the XM-pipeline showed marginal but statistically significant differences from the others: AUC 0.970 (95% CI 0.967–0.972) for XM-pipeline *versus* 0.966 (95% CI 0.961–0.971) for baseline (*p* < 0.001), 0.962 (95% CI 0.958–0.965) for HE (*p* < 0.001), 0.965 (95% CI 0.963–0.968 for CLAHE (*p* = 0.097), and 0.965 (95% CI 0.963–0.966) for UM (*p* = 0.002)). For the external test datasets, the performance of the AI model that utilized the XM-pipeline consistently outperformed those of the others.

**Table 2:** Diagnostic performance of the AI models with different pipelines

Test dataset	XM-pipeline	Baseline	HE	CLAHE	UM
	AUC (95% CI)	AUC (95% CI) (*p*)	AUC (95% CI) (*p*)	AUC (95% CI) (*p*)	AUC (95% CI) (*p*)
VH_DR1_	**0.970 (0.967–0.972)**	0.966 (0.961–0.971) (< 0.001)	0.962 (0.958–0.965) (< 0.001)	0.965 (0.963–0.968) (0.097)	0.965 (0.963–0.966) (0.002)
IH_DR2_	**0.948 (0.944–0.951)**	0.898 (0.887–0.909) (< 0.001)	0.934 (0.929–0.939) (< 0.001)	0.931 (0.927–0.935) (< 0.001)	0.914 (0.902–0.926) (< 0.001)
SZ_DR3_	**0.956 (0.951–0.960)**	0.908 (0.895–0.921) (< 0.001)	0.949 (0.944–0.954) (0.005)	0.933 (0.923–0.943) (< 0.001)	0.917 (0.901–0.932) (< 0.001)
IH_CR1_	**0.945 (0.940–0.950)**	0.899 (0.889–0.909) (< 0.001)	0.933 (0.925–0.941) (0.063)	0.920 (0.909–0.931) (< 0.001)	0.906 (0.887–0.924) (< 0.001)
IH_CR2_	**0.944 (0.939–0.948)**	0.658 (0.622–0.692) (< 0.001)	0.917 (0.908–0.926) (0.001)	0.705 (0.662–0.749) (< 0.001)	0.544 (0.520–0.567) (< 0.001)
MG_CR4_	**0.977 (0.974–0.981)**	0.918 (0.899–0.936) (< 0.001)	0.966 (0.957–0.974) (0.372)	0.958 (0.952–0.963) (0.038)	0.946 (0.922–0.970) (0.003)
IH_CR3,Mobile_	**0.949 (0.940–0.957)**	0.937 (0.927–0.947) (0.043)	0.933 (0.922–0.944) (0.042)	0.932 (0.923–0.942) (0.009)	0.925 (0.912–0.938) (0.001)

*AUC* Area under ROC curve, *CI* Confidence interval, *CLAHE* Contrast-limited histogram equalization, *HE* Histogram equalization, *UM* Unsharp masking

When investigating the results in more detail, the AI model with the XM-pipeline achieved better performance in all datasets acquired from CR systems (*i.e.*, IH_CR1_, IH_CR2_, IH_CR3,Mobile_, and MG_CR4_) compared to those of the other methods: *e.g.*, AUC in IH_CR2_ 0.944 (95% CI 0.939–0.948) for XM-pipeline *versus* 0.658 (95% CI 0.622–0.692) for baseline (*p* < 0.001), 0.917 (95% CI 0.908–0.926) for HE (*p* = 0.001), 0.705 (95% CI 0.662–0.749) for CLAHE (*p* < 0.001), and 0.544 (95% CI 0.520–0.567) for UM (*p* < 0.001), even if the model was trained using the data from a single DR system (*i.e.*, same data with VH_DR1_). In particular, in the IH_CR3,Mobile_ dataset acquired from a mobile x-ray machine, the AI model with the XM-pipeline outperformed the other models, reporting statistically significant differences: AUC 0.949 (95% CI 0.940–0.957) for XM-pipeline *versus* 0.937 (95% CI 0.927–0.947) for baseline (*p* = 0.043), 0.933 (95% CI 0.922–0.944) for HE (*p* = 0.042), 0.932 (95% CI 0.923–0.942) for CLAHE (*p* = 0.009), and 0.925 (95% CI 0.912–0.938) for UM (*p* = 0.001).

When we tested the AI models on the three large public datasets (CheXpert, ChestX-Det10, and RSNA-Pneumonia datasets), the AI model with the XM-pipeline outperformed the others. In the CheXpert dataset, the AI model with the XM-pipeline showed the best diagnostic performance: AUC 0.832 (95% CI 0.824–0.839) for XM-pipeline *versus* 0.822 (95% CI 0.809–0.835) for baseline (*p* < 0.001), 0.819 (95% CI 0.804–0.834) for HE (*p* < 0.001), 0.817 (95% CI 0.806–0.828) for CLAHE (*p* < 0.001), and 0.814 (95% CI 0.803–0.826) for UM (*p* = 0.001). In the ChestX-Det10 dataset, the AI model with the XM-pipeline reported the highest AUC: 0.920 (95% CI 0.916–0.924) for XM-pipeline *versus* 0.898 (95% CI 0.891–0.906) for baseline (*p* < 0.001), 0.913 (95% CI 0.907–0.920) for HE (*p* = 0.003), 0.909 (95% CI 0.903–0.915) for CLAHE (*p* = 0.001), and 0.899 (95% CI 0.891–0.907 (*p* = 0.001) for UM). In the RSNA-Pneumonia dataset, the AI model with the XM-pipeline also showed the best result: AUC 0.861 (95% CI 0.854–0.867) for XM-pipeline *versus* 0.854 (95% CI 0.842–0.866) for baseline (*p* < 0.001), 0.853 (95% CI 0.844–0.861) for HE (*p* = 0.001), 0.850 (95% CI 0.842–0.857) for CLAHE (*p* = 0.001), and 0.853 (95% CI 0.844–0.861) for UM, (*p* = 0.046).

**Fig. 5:**
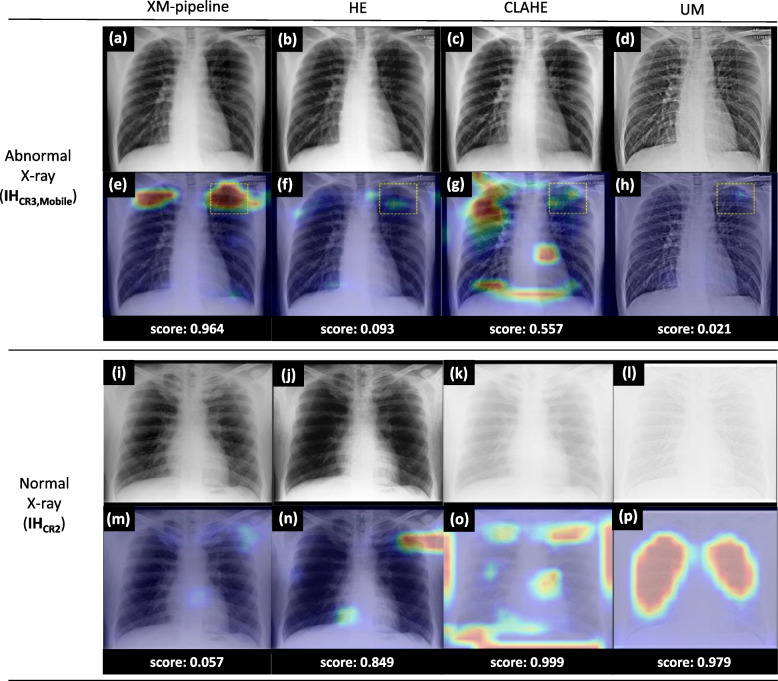
Analysis results of each AI model for the x-rays of selected patients. **a**–**d** and **i**–**l** Chest x-ray images after pre-processing. **e**–**h** and **m–p** Heatmaps and abnormality scores. In the abnormal chest x-ray image of a 31-year-old man (first row), the heatmap of the AI model with the XM-pipeline highlighted the exact region of fibrosis confirmed by a radiologist (yellow-dotted box in **e**) with the high abnormality score (= 0.964). In contrast, the others showed much smaller activations on these regions in the heatmaps (**e** *versus* **f**, **g**, **h**). Also, in the normal chest x-ray image of a 30-year-old man (second row), only the heatmap of the AI model with the XM-pipeline revealed no significant activations over the image (**m** *versus* **n**, **o**, **p**) with the minor abnormality score (= 0.057). *CLAHE* Contrast-limited histogram equalization, *HE* Histogram equalization, *UM* Unsharp masking

To further understand the behavior of the AI model, we generated heatmaps [43] for the x-rays of selected patients in the IH_CR2_ and IH_CR3,Mobile_ datasets (Fig. 5). In the heatmaps, the AI model with the XM-pipeline clearly highlighted abnormal regions compared to the other models.

### Stability of AI predictions

**Fig. 6:**
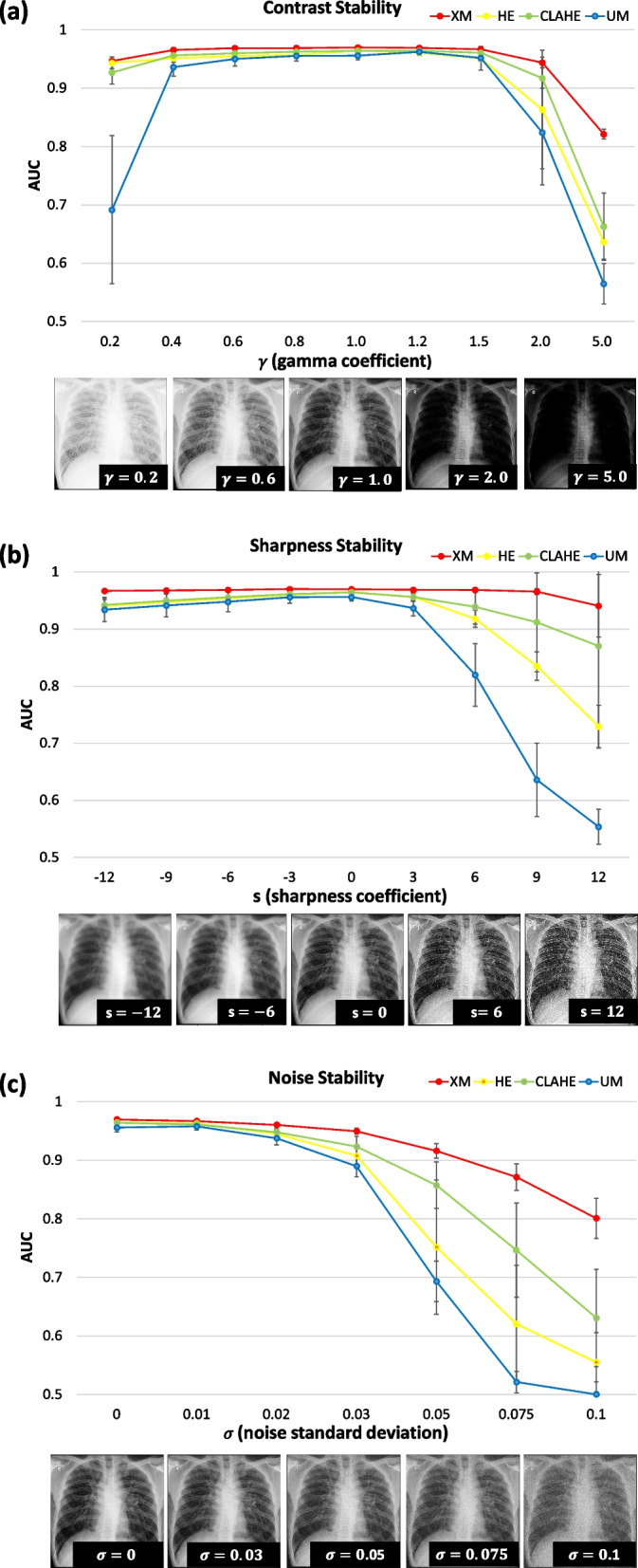
Diagnostic performance of each AI model depending on the changes in x-ray image characteristics. In general, the AI model with the XM-pipeline showed consistently higher diagnostic performance compared to those of the other models, such as after increasing the contrast (*e.g.*, AUCs when *γ* = 5.0: 0.821 (95% CI 0.814–0.829) for XM–pipeline, 0.636 (95% CI 0.610–0.667) for HE, 0.662 (95% CI 0.612–0.720) for CLAHE, and 0.565 (95% CI 0.534–0.599) for UM)) and increasing the sharpness (AUCs when *s* = 12: 0.960 (95% CI 0.951–0.971) for XM-pipeline, 0.729 (95% CI 0.572–0.908) for HE, 0.870 (95% CI 0.837–0.907) for CLAHE, and 0.553 (95% CI 0.526–0.584) for UM). *AUC* Area under ROC curve, *CLAHE* Contrast-limited histogram equalization, *HE* Histogram equalization, *UM* Unsharp masking

Figure 6 shows the diagnostic performance of each AI model depending on the changes in the image characteristics of input chest x-rays. When we changed the contrast of chest x-ray images (Fig. 6a), in general, the AI model trained using the XM-pipeline reported higher diagnostic performance compared to those of the other methods (*e.g.*, AUCs when $\gamma =5.0$: 0.821 (95% CI 0.814–0.829) for XM-pipeline, 0.636 (95% CI 0.610–0.667) for HE, 0.662 (95% CI 0.612–0.720) for CLAHE, and 0.565 (95% CI 0.534–0.599) for UM). When the contrast was low, UM especially showed degraded performance (*e.g.*, AUCs $\gamma =0.2$: 0.946 (95% CI 0.943–0.950) for XM-pipeline, 0.943 (95% CI 0.934–0.953) for HE, 0.927 (95% CI 0.909–0.947) for CLAHE, and 0.691 (95% CI 0.580–0.818) for UM). Similarly, when we changed the sharpness (Fig. 6b) and noise levels (Fig. 6c) of chest x-ray images, the AI model with the XM-pipeline demonstrated less degradation of the diagnostic performance, such as in cases of increasing sharpness (*e.g.*, AUCs when $s=12$: 0.960 (95% CI 0.951–0.971) for XM-pipeline, 0.729 (95% CI 0.572–0.908) for HE, 0.870 (95% CI 0.837–0.907) for CLAHE, and 0.553 (95% CI 0.526–0.584) for UM) and adding noise (*e.g.*, AUCs when $\sigma =0.1$: 0.801 (95% CI 0.771–0.835) for XM-pipeline, 0.555 (95% CI 0.510–0.606) for HE, 0.630 (95% CI 0.557–0.713) for CLAHE, and 0.500 (95% CI 0.481–0.521) for UM).

## Discussion

In this study, we proposed the XM-pipeline, which combines the series of image preprocessing and data augmentation methods to minimize the degradation of the diagnostic performance of an AI model for chest x-ray images of various machine specifications. We confirmed that the AI model with the XM-pipeline showed higher diagnostic performance than the other AI models with the conventional pipelines based on the test datasets of different x-ray machines, including CR or DR detectors and mobile or stationary generators.

In the XM-pipeline, we carefully designed the data augmentation techniques (see Supplementary Note 3 for detail) to consider the potential x-ray image variations depending on the x-ray scan settings (*e.g.*, changes of scan parameters, presence of grids, etc.) [44–46]. For example, due to the change in the voltage level of an x-ray generator, photons from the generator will have different amounts of energy. Accordingly, the contrast of chest x-ray images will be changed [47]. The sharpness in chest x-ray images can be changed depending on the presence of grids [48] and vendor-specific image processing techniques when producing DICOM [49]. Also, a chest x-ray image is known to have a mixture of noises due to several factors (*e.g.*, amount of radiation dose) [13], and we approximated the thermal and electronic noises by adding Gaussian noise to chest x-rays [50].

Previously, some studies have reported the diagnostic performance of AI models for chest x-ray images from multiple institutions [51, 52] and a few x-ray machines [53]. However, none of them proposed a training pipeline to improve the diagnostic performance and investigated AI models regarding different machine specifications, including the type of detectors and generators. Furthermore, we trained and evaluated the AI models using raw image data from DICOM files without adjusting the window level and width, while many open datasets provide x-ray data in Portable Network Graphics or Joint Photographic Experts Group formats [20, 54]. We believe deploying an AI model optimized for DICOM files is more practical in clinics.

Throughout this study, we have chosen the two most common data augmentation techniques (*i.e.*, random rotation and horizontal flipping) as the conventional ones. To find out the effect of other augmentations, we measured the diagnostic performance of AI models by applying different sets of augmentations, including random shearing (degree within [-15, 15]) and scaling (scaling factor within [0.8, 1.2]). However, no combination of augmentations was superior on the test datasets, even after adding more augmentations (see Supplementary Table S4).

When we investigated the diagnostic performance of AI for each abnormality, we found that some abnormalities were more challenging than others in terms of generalization (see Supplementary Table S5). For example, in the VH_DR1_ and IH_DR2_ datasets, the diagnostic performance for consolidation/ground glass opacity, pleural effusion, and pneumothorax were almost the same, while that for nodule/mass and fibrotic sequelae were degraded in IH_DR2_. However, the diagnostic performance was improved for all the abnormalities, after applying the XM-pipeline, compared to the baseline model.

This study has limitations. First, we could not fully explore the optimal parameters of the XM-pipeline (*e.g.*, range of sharpness coefficient). Therefore, the diagnostic performance can still be improved. Second, we adopted the most common image processing techniques for comparison, such as HE, CLAHE, and UM. However, other techniques exist to normalize chest x-ray images, such as [55]. Third, even if we carefully designed the data augmentation techniques in the XM-pipeline, those techniques are still limited to reflect the changes of image characteristics in chest x-rays. In the future, more realistic algorithms, such as the Monte Carlo method for x-ray scattering [56], can be explored as advanced data augmentation techniques. Fourth, we have validated the diagnostic performance of the AI models for classifying common pulmonary abnormalities, but other applications, such as COVID-19 detection [1], might be addressed as future work. Fifth, in this study, quality control of chest x-rays was only performed on private datasets. Other large open datasets, such as CheXpert [20], might need to be reviewed by radiologists to prevent potential labeling issues [57, 58].

In summary, the diagnostic performance of the AI model with the XM-pipeline was consistently higher than those of the other models with the conventional pipelines when they were evaluated on the test datasets of different x-ray machine specifications. This result implies that applying the XM-pipeline can minimize the performance degradation of the AI model due to the changes in x-ray machine specifications.

### Supplementary Information

**Additional file 1: Supplementary Table S1.** Summary of detailed information about the datasets used in our retrospective study. A single chest X-ray was acquired for each patient. **Supplementary Table S2.** Distribution of target abnormalities based on the radiologist’s annotation. **Supplementary Table S3.** Summary of metrics for the diagnostic performance of each AI model (sensitivity, specificity, positive predictive value, negative predictive value, and accuracy). **Supplementary Table S4. **Diagnostic performance of AI models by applying different combinations of data augmentation techniques. None of the combinations dominantly outperformed others for the test datasets. CLAHE was used for pre-processing. **Supplementary Table S5.** The diagnostic performance of the baseline and AI model with the XM-pipeline for each abnormality. If the number of chest X-rays for each abnormality is less than 30, we did not calculate the AUC values because of the small number of samples. **Supplementary Fig. S1.** Example chest X-ray images from each test dataset after applying the conventional pre-processing methods and the histogram modification in the XM-pipeline: original (first column), HE (second column), CLAHE (third column), UM (fourth column), and the histogram modification in the XM-pipeline (fifth column). The right upper zone (yellow-dotted box) of each image was zoomed in for investigation. **Supplementary Note 1.** Filtering Out X-ray Data on Large Public Datasets. **Supplementary Note 2.** Histogram Modification in XM-pipeline. **Supplementary Fig. S2.** Example images and histograms after applying each pre-processing step of the XM-pipeline. (a) Original chest X-ray image with its histogram. (b) Chest X-ray image after applying the iterative histogram clipping process. (c) Chest X-ray image after changing the minimum value of the histogram as the minimum intensity value inside the lung region. **Supplementary Note 3.** Data Augmentation in XM-pipeline. **Supplementary Fig. S3.** Example images with different γ values. (a) and (b): X-rays with γ value less than one. (c): original chest X-ray image. (d) X-rays with γ value greater than ones. **Supplementary Fig. S4.** Example images with different s values. **Supplementary Fig. S5.** Example image with different σ values.

## Data Availability

The datasets generated and/or analyzed during the current study are not publicly available due to privacy issues but are available from the corresponding author upon reasonable request.

## Abbreviations

AUC: Area under the curve at receiver operating characteristics analysis
CI: Confidence interval
CLAHE: Contrast-limited histogram equalization
CR: Computed radiography
DICOM: Digital imaging and communications in medicine
DR: Digital radiography
HE: Histogram equalization
UM: Unsharp masking

## References

[1] Keidar D, Yaron D, Goldstein E (2021). COVID-19 classification of x-ray images using deep neural networks. Eur Radiol.

[2] Lee JH, Park S, Hwang EJ (2021). Deep learning–based automated detection algorithm for active pulmonary tuberculosis on chest radiographs: diagnostic performance in systematic screening of asymptomatic individuals. Eur Radiol.

[3] Rajpurkar P, Irvin J, Zhu K, et al. (2017) CheXNet: radiologist-level pneumonia detection on chest x-rays with deep learning. arXiv preprint.10.48550/arXiv.1711.05225

[4] Nam JG, Park S, Hwang EJ (2019). Development and validation of deep learning-based automatic detection algorithm for malignant pulmonary nodules on chest radiographs. Radiology.

[5] Lakhani P, Sundaram B (2017). Deep learning at chest radiography: automated classification of pulmonary tuberculosis by using convolutional neural networks. Radiology.

[6] Li D, Pehrson LM, Lauridsen CA (2021). The added effect of artificial intelligence on physicians’ performance in detecting thoracic pathologies on CT and chest x-ray: a systematic review. Diagnostics.

[7] Kim EY, Kim YJ, Choi WJ (2022). Concordance rate of radiologists and a commercialized deep-learning solution for chest x-ray: real-world experience with a multicenter health screening cohort. PLoS One.

[8] Jones CM, Danaher L, Milne MR (2021). Assessment of the effect of a comprehensive chest radiograph deep learning model on radiologist reports and patient outcomes: a real-world observational study. BMJ Open.

[9] Seah JCY, Tang CHM, Buchlak QD (2021). Effect of a comprehensive deep-learning model on the accuracy of chest x-ray interpretation by radiologists: a retrospective, multireader multicase study. Lancet Digit Heal.

[10] Shin HJ, Han K, Ryu L, Kim EK (2023). The impact of artificial intelligence on the reading times of radiologists for chest radiographs. NPJ Digit Med.

[11] Schaefer-Prokop C, Neitzel U, Venema HW (2008). Digital chest radiography: an update on modern technology, dose containment and control of image quality. Eur Radiol.

[12] Kim YP, Park YP, Cheon MW (2018). A study on the characteristics of mobile x-ray device using supercapacitor as internal power. J Xray Sci Technol.

[13] Huda W, Abrahams RB (2015). Radiographic techniques, contrast, and noise in x-ray imaging. AJR Am J Roentgenol.

[14] Tommasi T, Patricia N, Caputo B, Tuytelaars T (2017) A deeper look at dataset bias. Domain Adapt Comput Vis Appl 37–55. 10.1007/978-3-319-58347-1_2

[15] Pooch EHP, Ballester P, Barros RC (2020) Can we trust deep learning based diagnosis? The impact of domain shift in chest radiograph classification. Paper presented at the International Workshop on Thoracic Image Analysis, Lima, Peru. 10.1007/978-3-030-62469-9_7

[16] López-Cabrera JD, Orozco-Morales R, Portal-Diaz JA (2021). Current limitations to identify COVID-19 using artificial intelligence with chest x-ray imaging. Health Technol.

[17] Cohen JP, Hashir M, Brooks R, Bertrand H (2020) On the limits of cross-domain generalization in automated x-ray prediction. Proceedings of the 3rd Conference on Medical Imaging with Deep Learning, Montreal, Canada

[18] Tkachenko M, Malyuk M, Holmanyuk A, Liubimov N (2022) Label studio: data labeling software. Available via https://labelstud.io/

[19] Jaeger S, Candemir S, Antani S (2014). Two public chest x-ray datasets for computer-aided screening of pulmonary diseases. Quant Imaging Med Surg.

[20] Irvin J, Rajpurkar P, Ko M, et al (2019) CheXpert: a large chest radiograph dataset with uncertainty labels and expert comparison. Proceedings of the 33rd AAAI Conference on Artificial Intelligence, Honolulu, Hawaii. 10.1609/aaai.v33i01.3301590

[21] Liu J, Lian J, Yu Y (2020) ChestX-Det10: chest x-ray dataset on detection of thoracic abnormalities. arXiv preprint. 10.48550/arXiv.2006.10550

[22] Shih G, Wu CC, Halabi SS, et al. (2019) Augmenting the national institutes of health chest radiograph dataset with expert annotations of possible pneumonia. Radiol Artif Intell 1. 10.1148/ryai.2019180041 10.1148/ryai.2019180041 PMC8017407 33937785

[23] Tan M, Le Q. (2019) EfficientNet: rethinking model scaling for convolutional neural networks. Proceedings of the 36th International Conference on Machine Learning, CA, USA.

[24] Kingma DP, Ba JL (2015) Adam: a method for stochastic optimization. arXiv preprint. 10.48550/arXiv.1412.6980

[25] Drummond C (2003). Holte RC (2003) Class imbalance, and cost sensitivity: why under-sampling beats over-sampling.

[26] Zamperoni P (1995). Image enhancement. advances in imaging and electron physics.

[27] Pizer SM, Amburn EP, Austin JD (1987). Adaptive histogram equalization and its variations. Comput graph image process.

[28] Polesel A, Ramponi G, Mathews VJ (2000). Image enhancement via adaptive unsharp masking. IEEE Trans Image Process.

[29] Munadi K, Muchtar K, Maulina N, Pradhan B (2020). Image enhancement for tuberculosis detection using deep learning. IEEE Access.

[30] Norval M, Wang Z, Sun Y (2019) Pulmonary tuberculosis detection using deep learning convolutional neural networks. Proceedings of the 3rd International Conference on Video and Image Processing, NY, USA. 10.1145/3376067.3376068

[31] Giełczyk A, Marciniak A, Tarczewska M, Lutowski Z (2022). Pre-processing methods in chest x-ray image classification. PLoS ONE.

[32] Chokchaithanakul W, Punyabukkana P, Chuangsuwanich E (2022). Adaptive image preprocessing and augmentation for tuberculosis screening on out-of-domain chest X-Ray dataset. IEEE Access.

[33] Rahman T, Khandakar A, Qiblawey Y (2021). Exploring the effect of image enhancement techniques on COVID-19 detection using chest x-ray images. Comput Biol Med.

[34] Abbas A, Abdelsamea MM (2018) Learning transformations for automated classification of manifestation of tuberculosis using convolutional neural network. Proceedings of the 13th International Conference on Computer Engineering and Systems, Cairo, Egypt. 10.1109/ICCES.2018.8639200

[35] Ahsan M, Gomes R, Denton A (2019) Application of a convolutional neural network using transfer learning for tuberculosis detection. Proceedings of the IEEE International Conference on Electro Information Technology, South Dakota, USA. 10.1109/EIT.2019.8833768

[36] Minaee S, Kafieh R, Sonka M (2020). Deep-COVID: predicting COVID-19 from chest x-ray images using deep transfer learning. Med Image Anal.

[37] Wang L, Lin ZQ, Wong A (2020). COVID-Net: a tailored deep convolutional neural network design for detection of COVID-19 cases from chest x-ray images. Sci Rep.

[38] Celik T (2014). Spatial entropy-based global and local image contrast enhancement. IEEE Trans Image Process.

[39] Somasundaram K, Kalavathi P (2011). Medical image contrast enhancement based on gamma correction. Int J Knowl Manag e-Learning.

[40] Dewangan S, Kumar Sharma A (2017). Image smoothening and sharpening using frequency domain filtering technique. Int J Emerg Technol Eng Res.

[41] Chlap P, Min H, Vandenberg N (2021). A review of medical image data augmentation techniques for deep learning applications. J Med Imaging Radiat Oncol.

[42] Sun X, Xu W (2014). Fast implementation of DeLong’s algorithm for comparing the areas under correlated receiver operating characteristic curves. IEEE Signal Process Lett.

[43] Ye W, Yao J, Xue H, Li Y (2020) Weakly supervised lesion localization with probabilistic-CAM pooling. arXiv preprint. 10.48550/arXiv.2005.14480

[44] Aichinger H, Dierker J, Joite-Barfuß S, Säbel M (2012). Radiation exposure and image quality in x-ray diagnostic radiology: physical principles and clinical applications.

[45] Thunthy KH, Manson-Hing LR (1978). Effect of mAs and kVp on resolution and on image contrast. Oral Surg Oral Med Oral Pathol.

[46] Sauter AP, Andrejewski J, Frank M (2021). Correlation of image quality parameters with tube voltage in x-ray dark-field chest radiography: a phantom study. Sci Rep.

[47] McKetty MH (1998). The AAPM/RSNA physics tutorial for residents: x-ray attenuation. Radiographics.

[48] Mazurov A, Potrakhov N (2015). Effect of scattered x-ray radiation on imaging quality and techniques for its suppression. Biomed Eng.

[49] Dance D, Christofides S, Maidment A, et al. (2014) Diagnostic radiology physics. Int At Energy Agency 299:183–193

[50] Lee S, Lee MS, Kang MG (2018). Poisson-gaussian noise analysis and estimation for low-dose x-ray images in the NSCT domain. Sensors.

[51] Hwang EJ, Park S, Jin KN (2019). Development and validation of a deep learning-based automated detection algorithm for major thoracic diseases on chest radiographs. JAMA Netw open.

[52] Jin KN, Kim EY, Kim YJ (2022). Diagnostic effect of artificial intelligence solution for referable thoracic abnormalities on chest radiography: a multicenter respiratory outpatient diagnostic cohort study. Eur Radiol.

[53] Govindarajan A, Govindarajan A, Tanamala S, et al. (2022) Role of an automated deep learning algorithm for reliable screening of abnormality in chest radiographs: a prospective multicenter quality improvement study. Diagnostics 12. 10.3390/diagnostics12112724 10.3390/diagnostics12112724 PMC9689183 36359565

[54] Wang X, Peng Y, Lu L, et al. (2017) Chestx-ray8: hospital-scale chest x-ray database and benchmarks on weakly-supervised classification and localization of common thorax diseases. Proceedings of the IEEE Conference on Computer Vision and Pattern Recognition, Honolulu, Hawaii. 10.1109/CVPR.2017.369

[55] Kim H, Lee S, Shim WJ (2023). Homogenization of multi-institutional chest x-ray images in various data transformation schemes. J Med Imaging.

[56] Lee H, Lee J (2019). A deep learning-based scatter correction of simulated x-ray images. Electronics.

[57] Oakden-Rayner L (2020). Exploring large-scale public medical image datasets. Acad Radiol.

[58] Garcia Santa Cruz B, Bossa MN, Sölter J, Husch AD (2021). Public COVID-19 x-ray datasets and their impact on model bias – a systematic review of a significant problem. Med Image Anal.

