# Supplementary material for: Pulmonary abnormality screening on chest x-rays from different machine specifications: a generalized AI-based image manipulation pipeline
Source: Eur Radiol Exp. 2023 Nov 9;7:68. doi: 10.1186/s41747-023-00386-1 (PMC10632317; doi:10.1186/s41747-023-00386-1)
Supplement: Supplementary file 1 — Additional file 1: Supplementary Table S1. Summary of detailed information about the datasets used in our retrospective study. A single chest X-ray was acquired for each patient. Supplementary Table S2. Distribution of target abnormalities based on the radiologist’s annotation. Supplementary Table S3. Summary of metrics for the diagnostic performance of each AI model (sensitivity, specificity, positive predictive value, negative predictive value, and accuracy). Supplementary Table S4. Diagnostic performance of AI models by applying different combinations of data augmentation techniques. None of the combinations dominantly outperformed others for the test datasets. CLAHE was used for pre-processing. Supplementary Table S5. The diagnostic performance of the baseline and AI model with the XM-pipeline for each abnormality. If the number of chest X-rays for each abnormality is less than 30, we did not calculate the AUC values because of the small number of samples. Supplementary Fig. S1. Example chest X-ray images from each test dataset after applying the conventional pre-processing methods and the histogram modification in the XM-pipeline: original (first column), HE (second column), CLAHE (third column), UM (fourth column), and the histogram modification in the XM-pipeline (fifth column). The right upper zone (yellow-dotted box) of each image was zoomed in for investigation. Supplementary Note 1. Filtering Out X-ray Data on Large Public Datasets. Supplementary Note 2. Histogram Modification in XM-pipeline. Supplementary Fig. S2. Example images and histograms after applying each pre-processing step of the XM-pipeline. (a) Original chest X-ray image with its histogram. (b) Chest X-ray image after applying the iterative histogram clipping process. (c) Chest X-ray image after changing the minimum value of the histogram as the minimum intensity value inside the lung region. Supplementary Note 3. Data Augmentation in XM-pipeline. Supplementary Fig. S3. Example images with di [file 41747_2023_386_MOESM1_ESM.docx]

**Pulmonary abnormality screening on chest x-rays from different machine specifications: a generalized AI-based image manipulation pipeline**

**ELECTRONIC SUPPLEMENTARY MATERIAL**

**Supplementary Table S1.** Summary of detailed information about the datasets used in our retrospective study. A single chest X-ray was acquired for each patient.


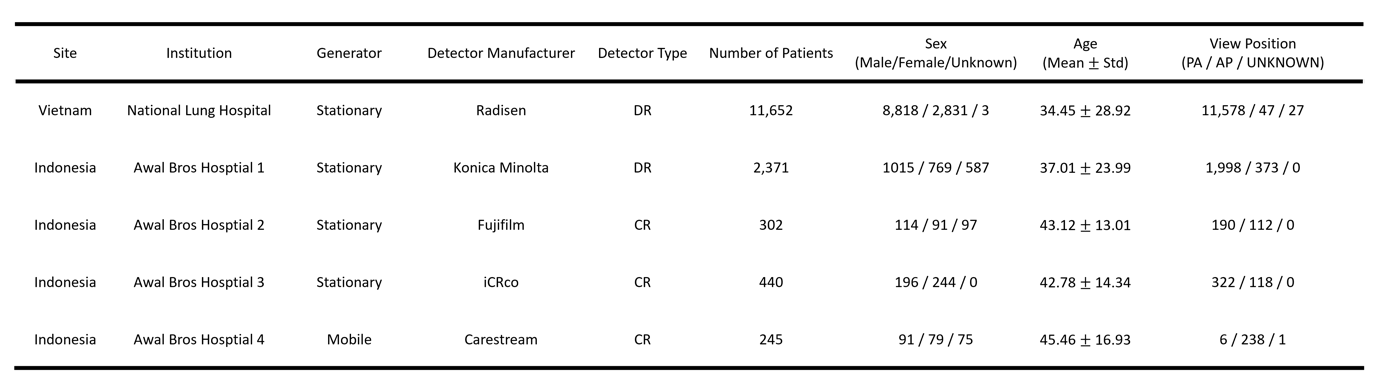


**Supplementary Table S2.** Distribution of target abnormalities based on the radiologist’s annotation.


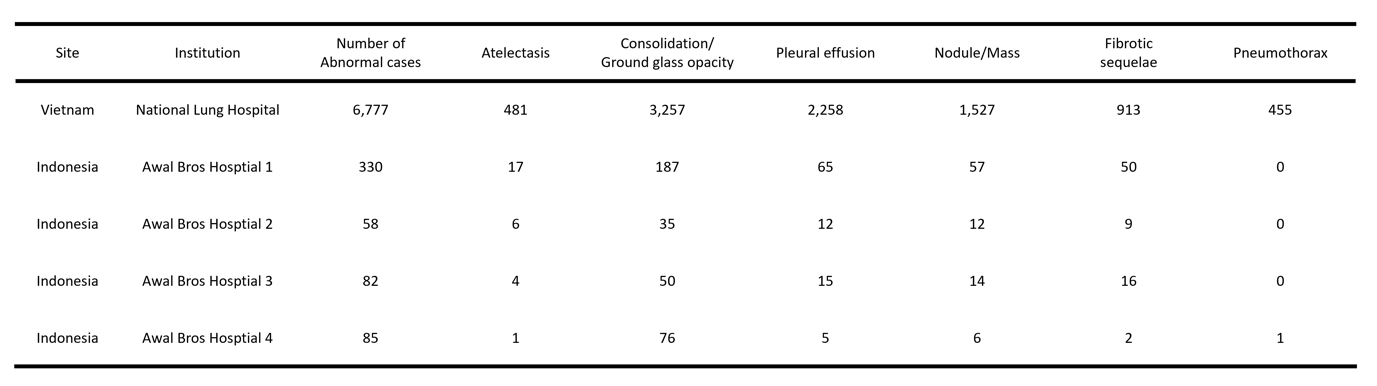


**Supplementary Table S3.** Summary of metrics for the diagnostic performance of each AI model (sensitivity, specificity, positive predictive value, negative predictive value, and accuracy).


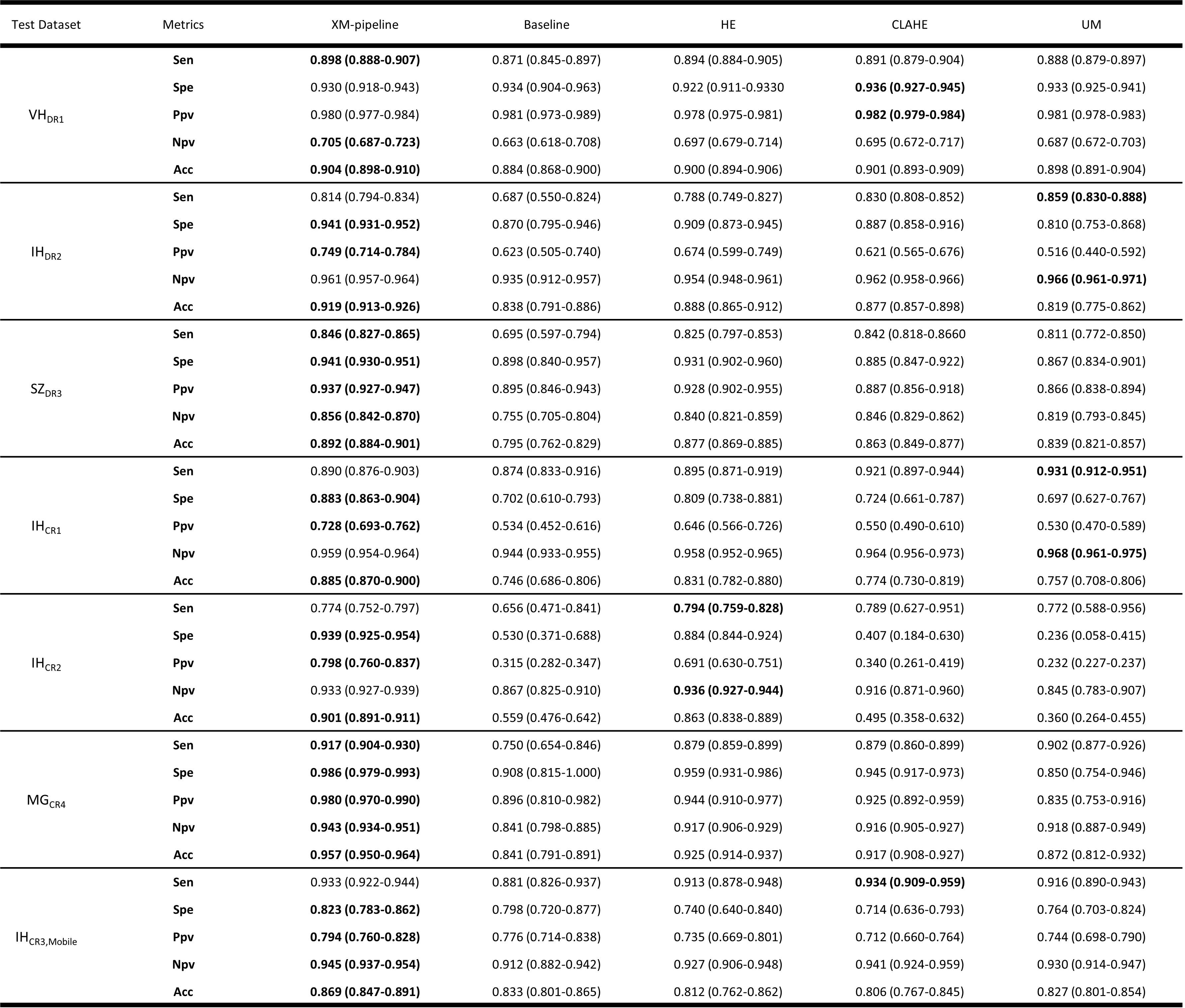


**Supplementary Table S4.** Diagnostic performance of AI models by applying different combinations of data augmentation techniques. None of the combinations dominantly outperformed others for the test datasets. CLAHE was used for pre-processing.


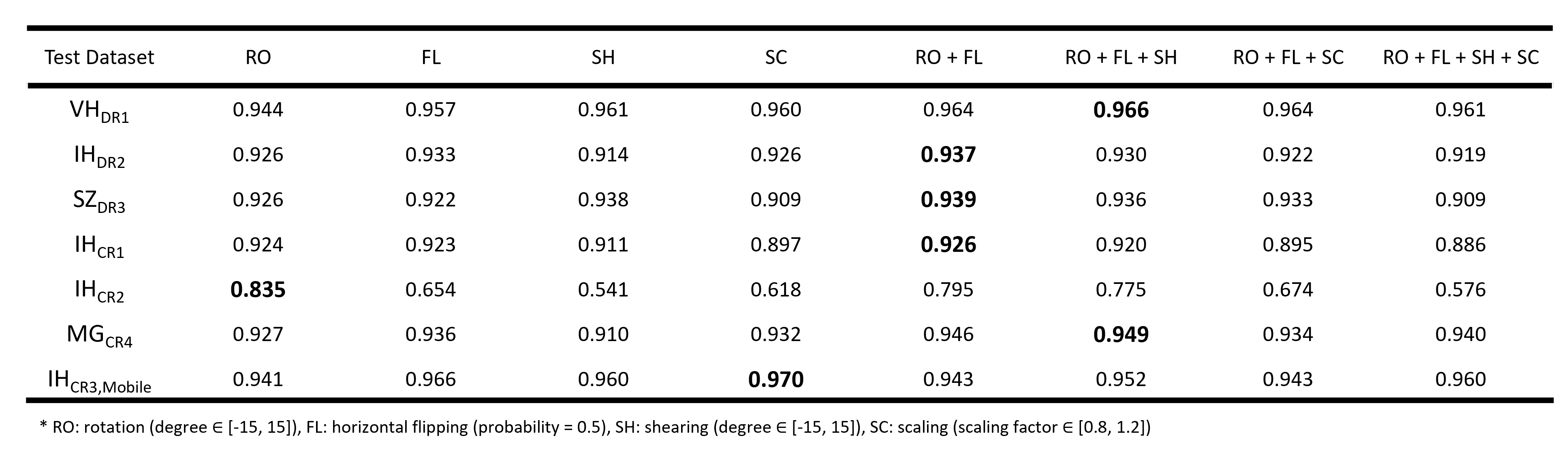


**Supplementary Table S5.** The diagnostic performance of the baseline and AI model with the XM-pipeline for each abnormality. If the number of chest X-rays for each abnormality is less than 30, we did not calculate the AUC values because of the small number of samples.


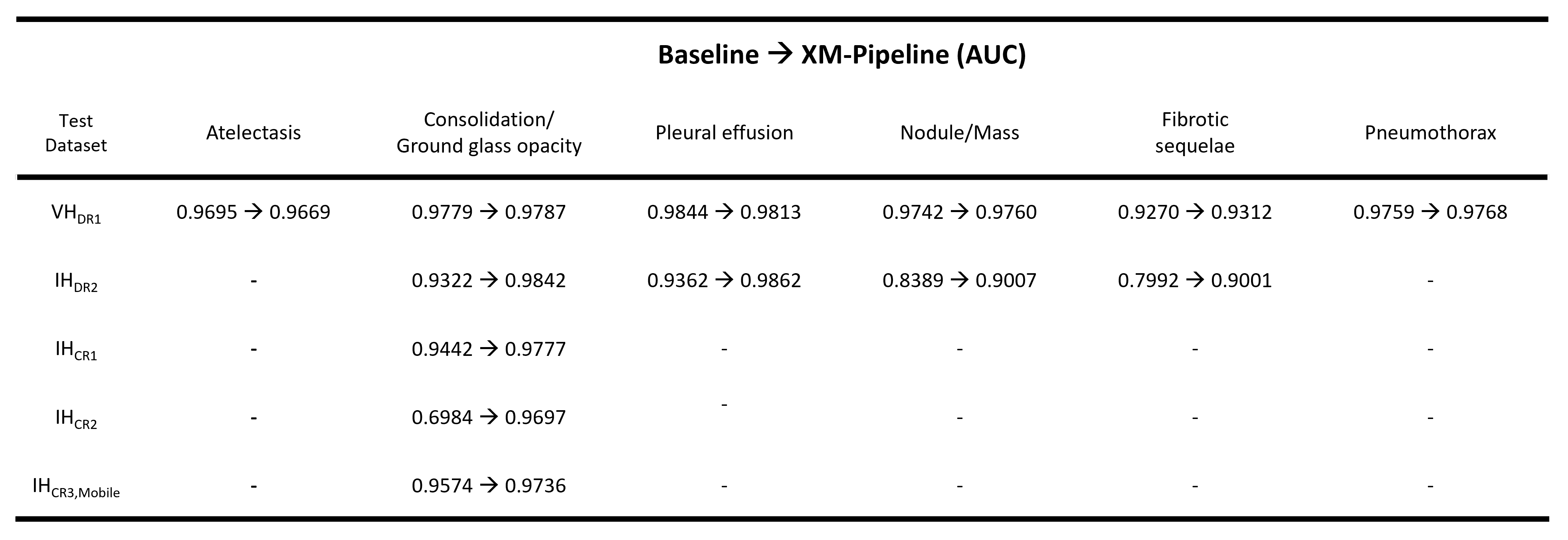


Supplementary Fig. S1. Example chest X-ray images from each test dataset after applying the conventional pre-processing methods and the histogram modification in the XM-pipeline: original (first column), HE (second column), CLAHE (third column), UM (fourth column), and the histogram modification in the XM-pipeline (fifth column). The right upper zone (yellow-dotted box) of each image was zoomed in for investigation.
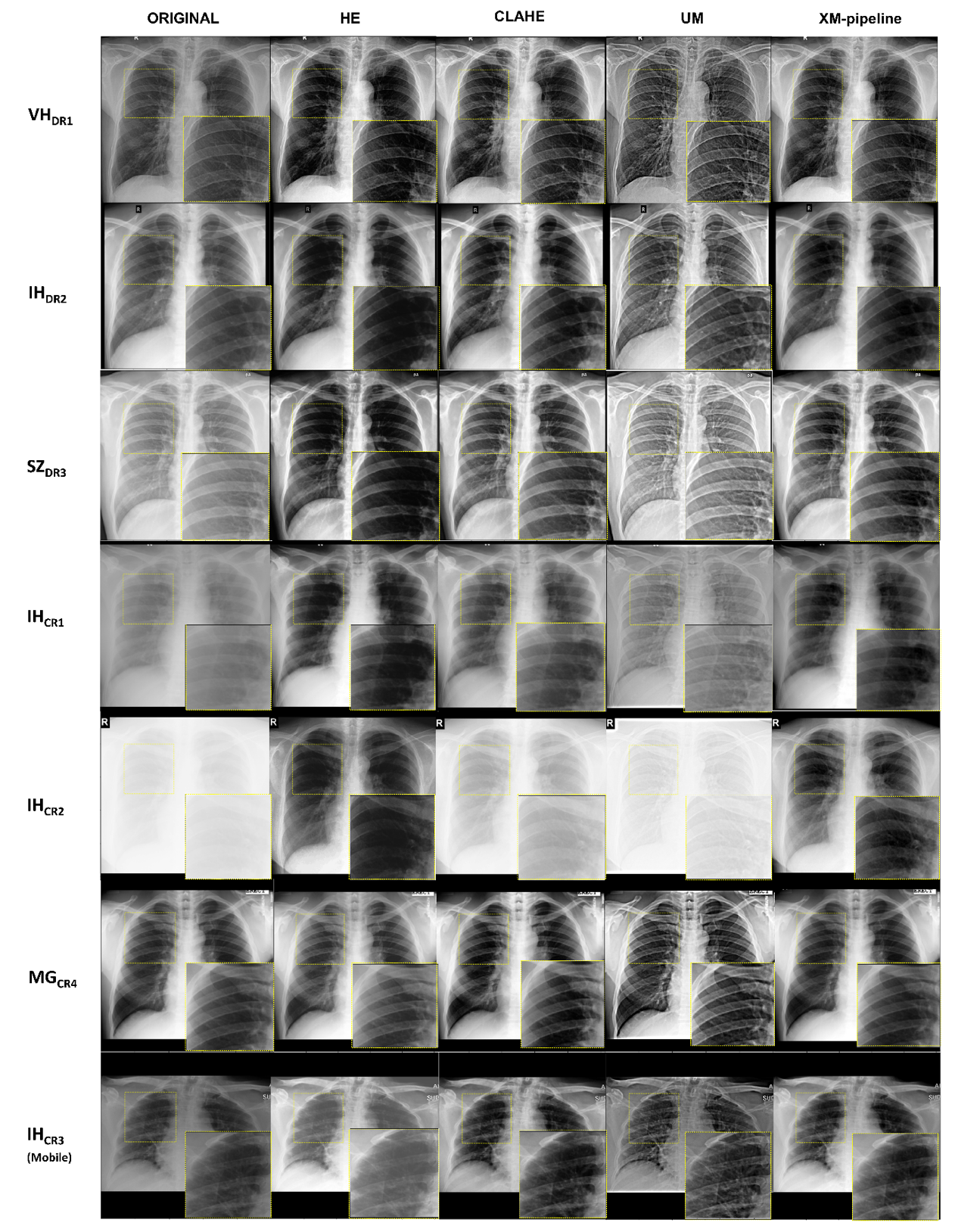


**Supplementary Note 1 – Filtering Out X-ray Data on Large Public Datasets**

We utilized three large public datasets for the additional evaluation of the AI models (CheXpert dataset [1], ChestX-Det10 dataset [2], and RSNA-Pneumonia dataset [3]). For those datasets, we randomly selected 500 normal (i.e., no findings) and 500 abnormal (i.e., at least one lung abnormality) X-ray images. In the CheXpert dataset, nine lung findings (lung opacity, lung lesion, edema, consolidation, pneumonia, atelectasis, pneumothorax, pleural effusion, and pleural other) were considered abnormal. In the ChestX-Det10 dataset, seven pulmonary abnormalities (atelectasis, consolidation, calcification, effusion, mass, nodule, and pneumothorax) were targeted.

**Supplementary Note 2 – Histogram Modification in XM-pipeline**

The pre-processing in the XM-pipeline aims to maximize the information inside the lung regions before AI training and testing. After this process, the contrast inside the lung regions will be maximized (see Supplementary Figure 2).

In many cases, chest X-ray images contain information about the patient’s orientation with symbols (e.g., ‘R’ and ‘AP’). These characters make the image’s body parts dark since the characters’ intensities are set as the maximum. To solve this problem, we iteratively clipped each histogram starting from the maximum intensity in the histogram and stretched it until reaching the maximum entropy, similar to [4] (see (a) vs. (b) in Supplementary Figure 2).

Since the pixel intensities of the background are significantly lower than those of the other body parts, this makes the lung field bright and limits the contrast inside the lung region. To address this issue, similar to [5], we set the minimum value of each histogram as the minimum intensity inside the lung field to enhance the contrast of soft tissues (see (b) vs. (c) in Supplementary Figure 2).

**Supplementary Fig. S2.** Example images and histograms after applying each pre-processing step of the XM-pipeline. (a) Original chest X-ray image with its histogram. (b) Chest X-ray image after applying the iterative histogram clipping process. (c) Chest X-ray image after changing the minimum value of the histogram as the minimum intensity value inside the lung region.


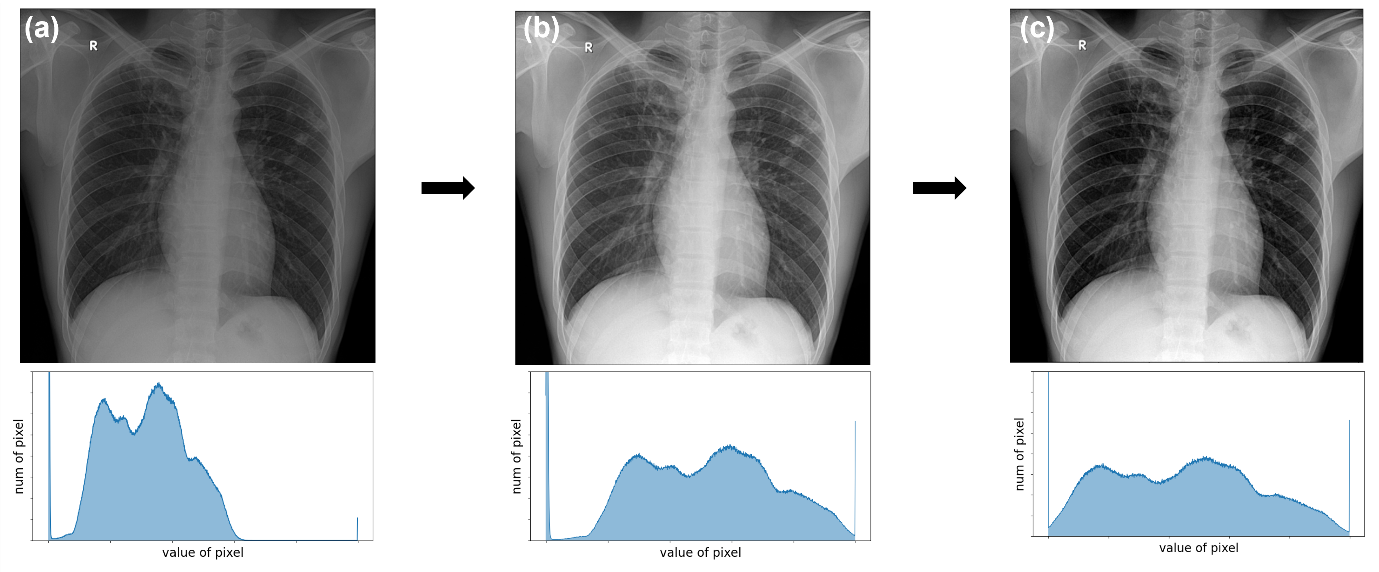


**Supplementary Note 3 – Data Augmentation in XM-pipeline**

1. Contrast Augmentation

To change the contrast of chest X-ray images, we adopted a gamma correction method [6] which utilizes a non-linear transformation of pixel intensity in an image as follows:

$$g\left( u\left( x,y \right) \right)={u(x,y)}^{\gamma}$$

where $g$ is a gamma correction function, $u\in[0,1]$ refers to a normalized pixel intensity, $x,y$ are coordinates in an image, and $\gamma$ is a pre-defined positive value. The $\gamma$ value greater than one increases the contrast of an X-ray image and vice versa. In this study, we empirically chose the maximum and minimum $\gamma$ values as 0.5 and 2.0 for AI training (see Supplementary Figure 3 for examples).

**Supplementary Fig. S3.** Example images with different $\gamma$ values. (a) and (b): X-rays with $\gamma$ value less than one. (c): original chest X-ray image. (d) X-rays with $\gamma$ value greater than ones.


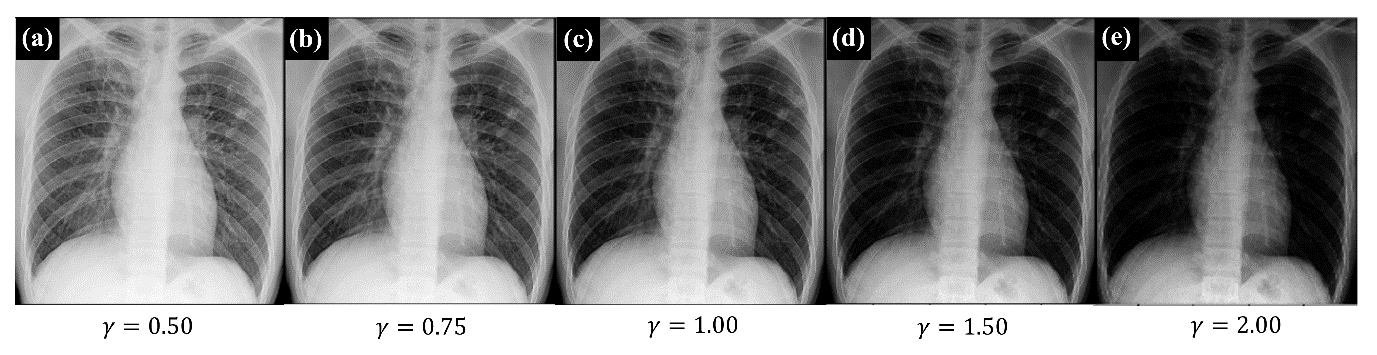


2. Sharpness Augmentation

To change the sharpness in chest X-ray images, we multiplied a Gaussian filter by a two-dimensional Fourier spectrum of a chest X-ray image as follows:

$$\tilde{F}\left( x,y \right)=F(x,y) \cdot H\left( x,y \right)$$

where $\tilde{F}$ is a Fourier spectrum of a chest X-ray after applying the augmentation, $x, y$ are coordinates in the spectrum, $F$ indicates a spectrum of an original chest X-ray image, and $H$ is a Gaussian filter.

To enhance or reduce the sharpness, we defined a Gaussian filter as follows:

$$H\left( x,y \right)=\left\{ \begin{aligned} e^{D^{2}\left( x,y \right)*s} when s<0 \\ 2- e^{-D^{2}\left( x,y \right)*s} when s>0 \end{aligned} \right.$$

where $D$ is the distance from the center point in a Fourier spectrum of a chest X-ray image, and $s$ is the sharpness coefficient. We empirically chose the minimum and maximum $s$ as -6 and 6 and randomly applied the augmentation for each chest X-ray image (see Supplementary Figure 4 for examples).

**Supplementary Fig. S4.** Example images with different $s$ values. (a) and (b): blurred images with $s=-6$ and $s=-3$. (c): original image. (d) and (e): sharpened images with $s=3$ and $s=6$.


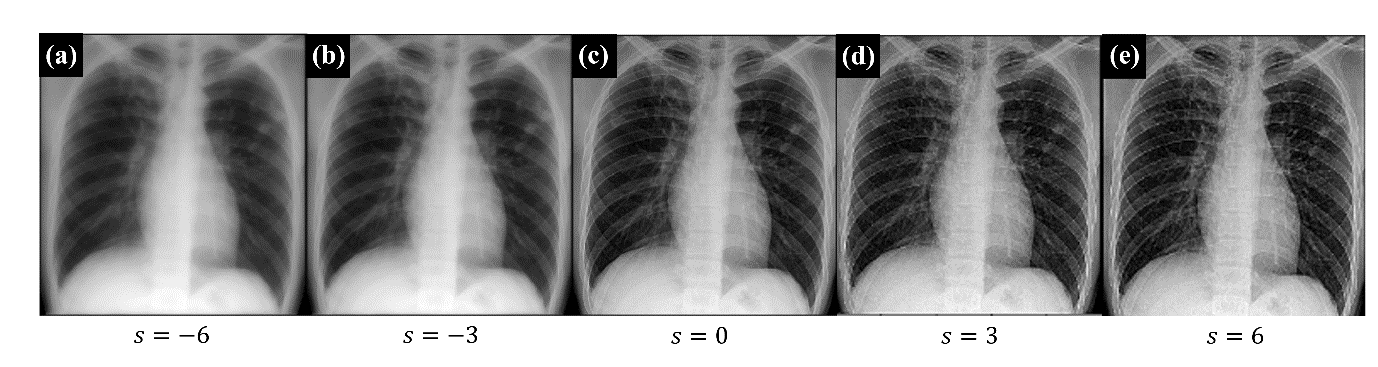


3. Noise Augmentation

To simulate the thermal and electronic noises in chest X-rays, we added Gaussian noise [7] as follows:

$$g\left( u(x,y) \right)=u(x,y)+ \sigma\cdot N(0,1)$$

where $g$ is pixel intensity after the augmentation, $u$ refers to each pixel in a chest X-ray image, $\sigma$ is a standard deviation of the noise, $N(0,1)$ is a standard normal distribution. We empirically set the minimum and maximum values of $\sigma$ as 0.01 and 0.1 for the augmentation (see Supplementary Figure 5 for examples).

**Supplementary Fig. S5.** Example image with different $\sigma$ values. (a): original image. (b), (c), and (d): noise injected image with $\sigma=0.03$, $\sigma=0.05$, and $\sigma=0.1$.


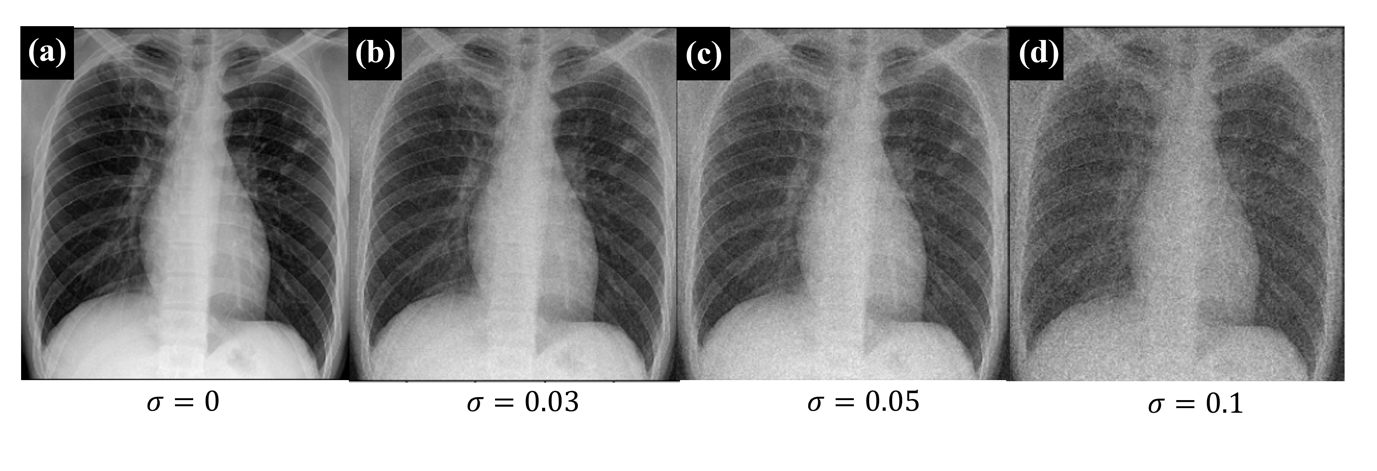


**References**

1. Irvin J, Rajpurkar P, Ko M, et al (2019) CheXpert: A large chest radiograph dataset with uncertainty labels and expert comparison. 33rd AAAI Conf Artif Intell AAAI 2019, 31st Innov Appl Artif Intell Conf IAAI 2019 9th AAAI Symp Educ Adv Artif Intell EAAI 2019 590–597. https://doi.org/10.1609/aaai.v33i01.3301590

2. Liu J, Lian J, Yu Y (2020) ChestX-Det10: Chest X-ray Dataset on Detection of Thoracic Abnormalities

3. Shih G, Wu CC, Halabi SS, et al (2019) Augmenting the national institutes of health chest radiograph dataset with expert annotations of possible pneumonia. Radiol Artif Intell 1:. https://doi.org/10.1148/ryai.2019180041

4. Celik T (2014) Spatial entropy-based global and local image contrast enhancement. IEEE Trans Image Process 23:5298–5308. https://doi.org/10.1109/TIP.2014.2364537

5. Chokchaithanakul W, Punyabukkana P, Chuangsuwanich E (2022) Adaptive Image Preprocessing and Augmentation for Tuberculosis Screening on Out-of-Domain Chest X-Ray Dataset. IEEE Access 10:132144–132152. https://doi.org/10.1109/ACCESS.2022.3229591

6. Somasundaram K, Kalavathi P (2011) Medical Image Contrast Enhancement Based ON Gamma Correction. Int J Knowl Manag e-Learning 3:15–18

7. Lee S, Lee MS, Kang MG (2018) Poisson-gaussian noise analysis and estimation for low-dose X-ray images in the NSCT domain. Sensors (Switzerland) 18:1–22. https://doi.org/10.3390/s18041019
